# Anti-HIV-1 antibodies based confirmatory results in Wuhan, China, 2012-2018

**DOI:** 10.1371/journal.pone.0238282

**Published:** 2020-09-11

**Authors:** Pan Liu, Li Tang, Wen-Hua Kong, Ze-Rong Zhu, Peng Xiao, Xia Wang, Wang Zhou, Man-Qing Liu

**Affiliations:** Wuhan Center for Disease Control & Prevention, Wuhan, Hubei, China; Indiana University School of Medicine, UNITED STATES

## Abstract

The number, intensity and order of emergence of HIV-1 specific antibodies in serum or plasma were associated with the stage of HIV-1 infection. In this study, we retrospectively analyzed the HIV-1 confirmatory results tested by western blot (WB) or recombination immunoblot assay (RIBA) in Wuhan, 2012–2018, to access the profiles of HIV-1 specific antibodies. A total of 14432 HIV-suspected serum or plasma samples collected from local hospitals and other HIV screening laboratories were further screened by two 4^th^ generation enzyme-linked immunosorbent assay (ELISA) kits in our laboratory, of which 11068 specimens (76.69%) had at least one positive ELISA result and thereby were finally confirmed with WB or RIBA. RIBA had identified 652 (81.09%) positive and 13 (1.62%) indeterminate cases from July 1, 2014 to January 7, 2015, while WB had identified 8358 (81.43%) positive and 643 (6.26%) indeterminate cases in the other times during 2012–2018. The indeterminate rate of WB was significant higher than that of RIBA (*p*<0.001). Although the number of HIV-1 infected subjects increased significantly from 2012 (n = 911) to 2018 (n = 1578), the positive rate of HIV-1 antibodies decreased markedly from 70.08% in 2012 to 58.79% in 2018 (*p*<0.001). The most commonly observed antibody profile was gp160+gp120+p66+(p55+)p51+gp41+p31+p24+p17+ (4131, 49.43%) for WB-MP and gp160+gp120+gp41+p31+p24+p17+ (382, 58.59%) for RIBA-WANTAI, and the absence of reactivity to three possible serologic markers for recent HIV-1 infection, p31, p66, and p51, increased significantly from 2012 to 2018, with the overall rate of 17.03%, 9.40%, and 15.15%, respectively. The suspected acute HIV-1 infection was also observed to be increased in recent years, with an overall rate of 1.00%. Our results indicated the detection rate had decreased for HIV-1 infection, but increased for suspected recent and acute HIV-1 infection during 2012–2018, reflecting the efforts of intervention among high risk population.

## Introduction

Human immunodeficiency virus (HIV) infection is a major global health concern and social problem [1]. Actions have been taken aimed to end AIDS in 2030 with the UNAIDS 90-90-90 target [2], however, providing HIV test to more than 90% of people living with HIV is a huge challenge. Efforts should be acknowledged, as increased acute/recent HIV infection has been identified in clinic in recent years [3]. Although more sensitive methods such as nucleic acid test (NAT) were recommended, western blotting (WB) remained to be mostly used as a gold standard method world widely [4] with high sensitivity and specificity for the detection of anti-HIV antibodies [5]. In order to reduce the rate of indeterminate results, line immuno assays (LIA) and recombination immunoblot assay (RIBA) were recruited as a supplemental confirmatory methods for HIV infection [6]. A study conducted in China has proved that RIBA could reduce the window period compared with WB, thus was recommended as a supplemental assay for confirming HIV infection in China [7]. Although RIBA has comparable performance to WB [8], it was more popularly used in the diagnosis of hepatitis C virus (HCV), but not HIV, which may due to the reason that HCV is extremely difficult to be tissue culture propagated [9] and thus can’t be used with natural virus particles for manufacturing WB kit.

The emergence of HIV antibodies presents the infection status. Studies had proved that the number, intensity, and the order of appearance of HIV antibodies in blood were associated with the stage of HIV-1 infection [10, 11]. Recently, we had estimated the seroconversion time of HIV-1 antibodies from a clinical observation and found that both p31 and p66 antibodies were potential serologic biomarkers of recent HIV-1 infection [12]. However, the rate of recent HIV-1 infection with absence of p31 or p66 in Wuhan was yet to be determined. Immunoglobulin G (IgG)- enzyme immunoassay (BED-CEIA), a method based on measuring the proportion of HIV-1-specific IgG to total IgG after seroconversion [13, 14], was the most evaluated assay for HIV incidence determination [15]. Studies had reported the rates of recent HIV-1 infection tested by BED-CEIA were 28.6% in Tianjin city [16], 16.14% in Sichuan province [17], and less than 1% in Liangshan prefecture [18], China, which were inconsistent in different areas. Although BED-CEIA was able to distinguish recent infection from long-term HIV-1 infections [15, 19], it was affected by CD4^+^T cell counts, HIV-1 viral loads, antiretroviral treatment (ART), and HIV-1 subtype [11]. Thus, bands-based methods such as WB and RIBA might be better to differentiate recent HIV-1 infection from established infection than BED-CEIA [11]. In this study, we retrospectively analyzed the band patterns of individuals with positive and indeterminate WB/RIBA results, and estimated the rate of recent infection in Wuhan, China.

## Materials and methods

### HIV screening and confirmation

From 2012 to 2018, the plasma or serum specimens with suspected HIV infection were collected from the district centers for disease prevention and control, hospitals, or other health screening centers in Wuhan. All specimens were initially screened by two fourth generation enzyme-linked immunosorbent assay (ELISA) kits in our laboratory, and the specimens with at least one reactive result of ELISA were finally confirmed by WB (HIV BLOT 2.2, MP Diagnostics, Singapore) or RIBA (WANTAI BioPharm, China). The HIV antibody detection strategy and procedure of the confirmatory test were strictly in accordance with the National Guideline for Detection of HIV/AIDS (2015 revised edition) [7]. Interpretation of positive result was made according to the manufacturer’s instructions. For WB-MP kit, a positive result required the presence of at least three bands, including two *env* bands (gp160, gp120/gp41) plus one *gag* (p55, p24, p17) /*pol* (p66, p51, p31) band. RIBA-WANTAI kit was targeted for HIV-1/2 antibodies gp160, gp120, gp41, p31, p24, p17, and gp36, and a positive result for RIBA-WANTAI required at least two *env* bands, or one *env* band plus p24 band. Specimens that exhibited HIV-1 bands (except p17) yet did not qualify for the minimum criteria for positive result were defined as indeterminate, and a negative result was the absence of any of the specific bands (except p17). The WB or RIBA bands were visually verified by at least two experts independently. Subjects with negative HIV-1 WB/RIBA results but highly reactive for both 4^th^ generation ELISA kits were defined as suspected acute HIV-1 infection.

Written informed consent was obtained for the HIV test at the time of blood collection, and the Institutional Review Board of Wuhan CDC approved the study.

### Data collection

The demographic information of suspected HIV infection subjects and their laboratory test results for HIV-1/2 were recorded in Wuhan HIV Management Database. The anti-HIV antibody profiles as well as the demographic information were collected from the database for the subjects with HIV-1 positive and indeterminate results.

### Statistical analysis

Statistical analyses were performed with Prism (GraphPad Software, San Diego, CA). Chi-square test was used to compare the detection rates and a *p*-value <0.05 was considered as statistically significant.

## Results

### Basic information

**Fig 1:**
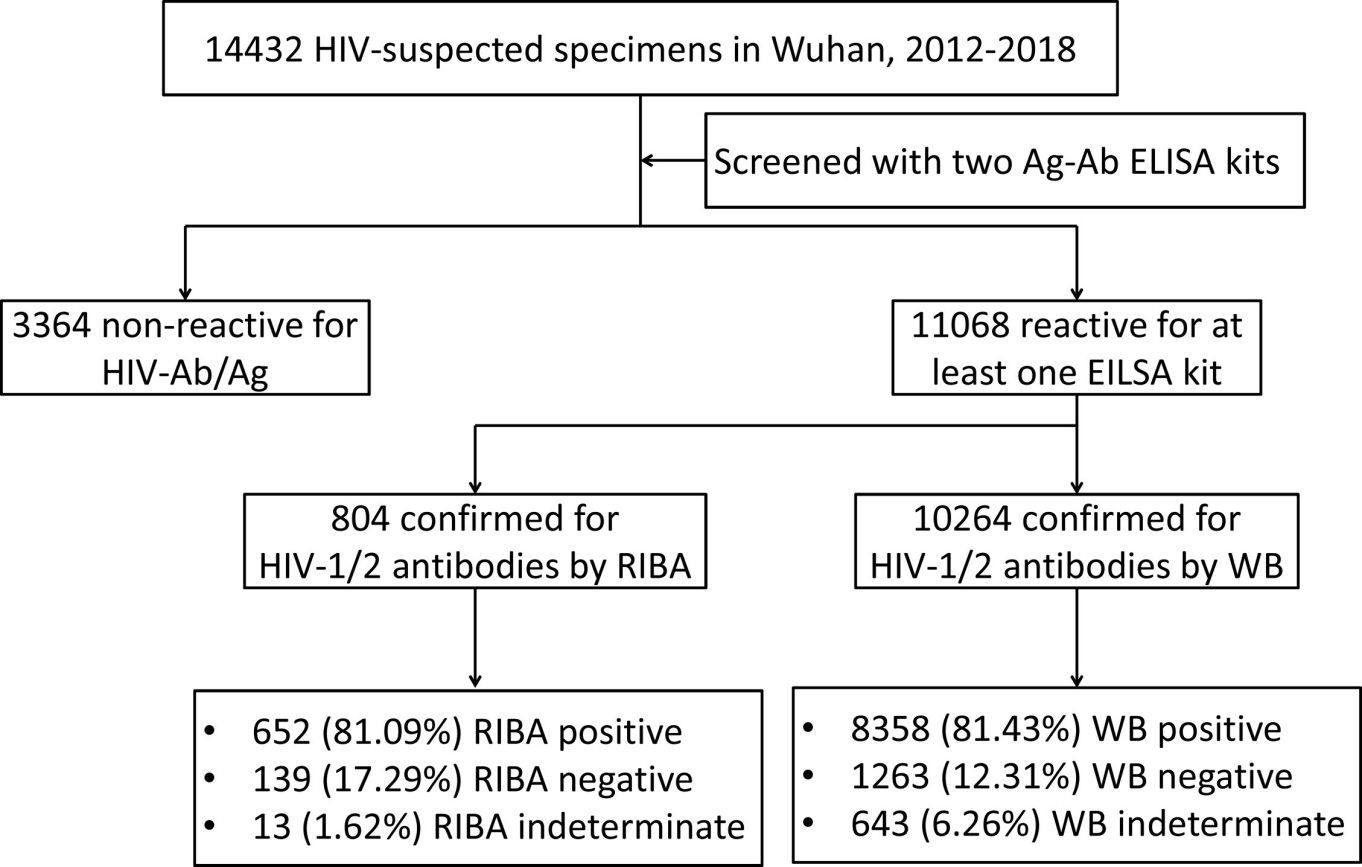
Flowchart of data collection for the comparison of WB and RIBA tested for HIV-1 infection in Wuhan, 2012–2018. Due to the unexpected procurement result, RIBA-WANTAI was used for HIV confirmatory test from July 1, 2014 to January 7, 2015, and WB-MP was used for other times. Two HIV confirmatory tests were significantly different in HIV detection rate, while WB had identified more HIV-1 indeterminate specimens than RIBA. Abbreviations: HIV, Human immunodeficiency virus; WB, western blot; RIBA, recombination immunoblot assay; ELISA, enzyme-linked immunosorbent assay; Ab, antibody; Ag, antigen.

A total of 14,432HIV-suspected specimens were collected from 2012 to 2018. After screened with two 4^th^ generation enzyme-linked immunosorbent assay (ELISA) kits, 11068 specimens (76.69%) were reactive in at least one ELISA test and were subjected to confirmatory test (WB or RIBA). Because of unexpected procurement, there were 804 specimens tested with RIBA-WANTAI from July 1, 2014 to January 7, 2015, of which 652 (81.09%) specimens were identified as HIV-1 positive, 139 (17.29%) as HIV-1 negative, and 13 (1.62%) as HIV-1 indeterminate. The other specimens (10264) were tested with WB-MP, of which 8358 subjects (81.43%) were determined as HIV-1 positive, 1263 (12.31%) as HIV-1 negative, and 643 (6.26%) as HIV-1 indeterminate (Fig 1). Among these WB or RIBA-negative cases, 144 specimens (144/14432, 1.00%) were highly reactive in the two 4^th^generation ELISA tests and thus were suspected to be acute HIV infection (Table 1). The HIV-1 positive rate was markedly decreased from 70.08% in 2012 to 58.79% in 2018 (*p*<0.0001), while the rate of HIV-1 indeterminate ranged from 2.81% to 5.89%. The majority of HIV-1 positive subjects were male (88.27%) and the largest age group was 21 to 30 years (33.87%), which may associate with the increased men who have sex with men (MSM) population in Wuhan.

**Table 1:** Characteristics of subjects with HIV-1 antibodies positive or indeterminate in Wuhan, China, 2012–2018.

Characteristics		Subjects with HIV-1 antibodies positive (n, %)	Subjects with HIV-1 antibodies indeterminate (n, %)
n		9010 (62.43%)	656 (4.55%)
Year			
	2012	911 (70.08%)	59 (4.54%)
	2013	975 (66.92%)	66 (4.53%)
	2014	1258 (64.25%)	55 (2.81%)
	2015	1262 (61.41%)	121 (5.89%)
	2016	1428 (61.47%)	117 (5.04%)
	2017	1598 (60.17%)	118 (4.44%)
	2018	1578 (58.79%)	120 (4.47%)
Sex			
	Male	7953 (88.27%)	517 (78.81%)
	Female	1053 (11.69%)	139 (21.19%)
	Unknown	4 (0.04%)	-
Age (years)			
	≤10	18 (0.20%)	11 (1.68%)
	11–20	486 (5.39%)	57 (8.69%)
	21–30	3052 (33.87%)	230 (35.06%)
	31–40	1705 (18.92%)	105 (16.01%)
	41–50	1649 (18.30%)	95 (14.48%)
	51–60	1248 (13.85%)	84 (12.80%)
	≥60	846 (9.39%)	72 (10.98%)
	Unknown	6 (0.07%)	2 (0.30%)

### HIV-1 antibodies positive specimens

During 2012–2018, a total of 9010 specimens were identified as HIV-1 positive. Although the number of HIV-1 positive specimens was increased, the positive rate was decreased in recent years (Table 1). Further analysis of HIV antibody profiles indicated that the most popular profile of WB-MP was gp160+gp120+p66+(p55+)p51+gp41+p31+p24+p17+ (4131, 49.43%), and that of RIBA-WANTAI was gp160+gp120+gp41+p31+p24+p17+ (382, 58.59%). Considering the bands of WB or RIBA may reflect the disease process, we further analyzed the absence rate of p31, p66, and p51. Table 2 showed that there were 1534 (17.03%) specimens with the absence of reactivity to p31 antibody, 786 (9.40%) specimens with the absence of reactivity to p66 antibody and 1266 (15.15%) specimens with the absence of reactivity to p51 antibody. The differences among the absence rate of p31, p66, and p51 were significant (*p*<0.001).

**Table 2:** Recent HIV-1 infection with the absence of band p31, p66, p51 and suspected acute HIV-1 infection in Wuhan city, 2012–2018.

Year	HIV-1 positive cases	Recent HIV-1 infection			Suspected acute HIV-1 infection^#^
		p31-	p66-	p51-	
2012	911 (70.08%)	111 (12.18%)	40 (4.39%)	106 (11.64%)	3 (0.23%)
2013	975 (66.92%)	132 (13.54%)	77 (7.90%)	114 (11.69%)	6 (0.41%)
2014	1258 (64.25%)	232 (18.44%)	122 (19.81%)*	158 (25.65%)*	10 (0.51%)
2015	1262 (61.41%)	258 (20.44%)	103 (8.23%)*	145 (11.58%)*	33 (1.61%)
2016	1428 (61.47%)	246 (17.23%)	140 (9.80%)	205 (14.36%)	31 (1.33%)
2017	1598 (60.17%)	269 (16.83%)	159 (9.95%)	283 (17.71%)	33 (1.24%)
2018	1578 (58.79%)	286 (18.12%)	145 (9.19%)	255 (16.16%)	28 (1.04%)
Total	9010 (62.43%)	1534 (17.03%)	786 (9.40%)*	1266 (15.15%)*	144 (1.00%)
X^2^	70.70	37.16	110.6	93.16	-
*p*	<0.0001	<0.0001	<0.0001	<0.0001	-

*There were 642 specimens in 2014 and 10 specimens in 2015 identified as anti-HIV-1 antibodies positive by RIBA from July 1, 2014 to January 7, 2015, which were not being tested for HIV antibodies p66 and p51 and therefore were excluded from the analysis. There were significant differences (p<0.001) among the absence rate for p31, p66, and p51 in the HIV-1 positive specimens collected from 2012 to 2018.

^#^Subjects with negative HIV-1 WB/RIBA results but highly reactive for both 4^th^ generation enzyme-linked immunosorbent assay (ELISA) kits were defined as suspected acute HIV-1 infection.

### HIV-1 antibodies indeterminate specimens

There were 656 specimens identified as HIV-1 antibodies indeterminate during 2012–2018, giving the overall indeterminate rate of 4.55%. The lowest indeterminate rate occurred in 2014, because RIBA was employed routinely in that year. Most indeterminate specimens came from men (78.81%) and as high as 35.06% occurred in individuals aged 21 to 30-years, followed by 16.01% occurred in individuals aged 31 to 40-years (Table 1). It was interesting that the proportion of female HIV-1 indeterminate cases was higher than female HIV-1 positive cases (*p*<0.001, 21.19% vs. 11.69%).

### Comparison of the confirmatory results between RIBA and WB

Our data indicated some differences on the confirmatory results between RIBA and WB, thus we further compared the detection rates and HIV-1 antibody profiles between these two confirmatory assays. Table 3 listed three most frequent band patterns for WB and RIBA methods, respectively. Considering that RIBA did not detect anti-p66, p55, and p51 antibodies, it showed similar HIV-1 antibody profiles to WB for both positive and indeterminate specimens regardless of bands p66, p55, p51. However, the constituent ratio of band patterns for HIV-1 positive specimens was different between WB and RIBA (χ^2^ = 70.72, *p*<0.0001), and WB showed higher indeterminate rate than RIBA (6.26% vs. 1.62%, *p*<0.001).

**Table 3:** Top 3 of the antibodies patterns tested by western blot (WB) or recombination immunoblot assay (RIBA) for the subjects with positive or indeterminate HIV-1 results in Wuhan city, 2012–2018.

Anti-HIV-1 results		Western Blot (WB)		Recombinant immunoblot assay (RIBA)	
		Pattern	n (%)	Pattern	n (%)
Positive	Top 1	gp160 gp120 p66 (p55) p51 gp41 p31 p24 p17	4131 (49.43%)	gp160 gp120 gp41 p31 p24 p17	382 (58.59%)
	Top 2	gp160 gp120 p66 (p55) p51 gp41 p31 p24	2136 (25.56%)	gp160 gp120 gp41 p31 p24	120 (18.40%)
	Top 3	gp160 gp120 p66 (p55) p51 gp41 p24	287 (3.43%)	gp160 gp41 p24	53 (8.13%)
Indeterminate*	Top 1	p24	169 (26.28%)	gp160 gp41	5 (38.46%)
	Top 2	gp160 gp41	153 (23.79%)	p24	3 (23.08%)
	Top 3	gp160	83 (12.91%)	gp41	3 (23.08%)

* WB showed significant higher detection rate of HIV-1 indeterminate than RIBA (6.26% vs. 1.62%, p<0.0001).

## Discussion

Band-based HIV antibody confirmatory assays such as WB and RIBA remained to be the mostly used strategy worldwide [20]. They are able to detect HIV specific antibodies in most subjects, but acute HIV infection and late-stage HIV infection maybe negative or indeterminate for HIV antibody tests [3, 21–23]. Previous studies have proven that the sequential emergence of HIV-1 antibodies associates with the progress of HIV-1 infection [11, 12, 24–26], therefore the laboratory diagnosis of HIV infection is helpful for understanding the local epidemic and may partly reflect the effectiveness of HIV/AIDS intervention. Our data indicated that, although the number of suspected samples gradually grown in Wuhan, the positive rate of HIV-1 antibodies has declined significantly during 2012–2018. In the same time, the recent HIV-1 infection and suspected acute HIV infection seem to be increased, which may be caused by the extensive usage of the sensitive 4^th^ generation ELISA and/or chemiluminescence immunoassay (CLIA) kits in China, and may also be associated with the efforts of China in intervening HIV infection among high risk population in recent years [27, 28]. Previous studies have reported that the number, intensity and order of emergence of WB bands were affected by the stage of HIV infection [10, 11], and *pol* antibodies may be predictors of seroconversion [4], especially p31 [10, 20, 29–31] and p66 [12]. In this study, we identified that the absence rates of p31, p66, and p51 were 17.03%, 9.40%, and 15.15% respectively and all of them increased significantly from 2012 to 2018. The finding is in agreement with other publications that more and more acute/recent HIV infection cases were found in recent years [3, 21] and implied the efforts in finding HIV infection by intervention in high risk population, one of the 2014 UNAIDS 90-90-90% targets for 2020 target. Most subjects with positive and indeterminate HIV-1 antibodies were male and aged between 21–30 years, which may due to current HIV epidemic in China that men who have sex with men (MSM) became the most popular transmission route of HIV infection [32, 33].

RIBA detects HIV-1 antibodies on the base of the recombinant DNA-derived HIV-1 antigens and is considered to be more sensitive and specific than methods based on native virus such as WB [8, 34]. Recent study has found that RIBA can reduce the window period to confirm the early HIV infection [6]. However, WB is more widely used for laboratory diagnosis of HIV infection than RIBA in practice. In this study, we compared the detection of HIV-1 by WB and RIBA, and found that they showed similar positive rates for HIV-1 specific antibodies, yet different in indeterminate rates, which could be caused by the different criteria for HIV-1 positive. However, due to nonspecific response [8, 35], indeterminate result tested by WB was significantly higher than that of RIBA and thereby was a major problem for HIV detection in clinical diagnosis [8].

### Limitations

Several limitations must be acknowledged. First, anti-HIV antibodies positive were defined according to the manufacturer’s instructions, which seemed stricter than World Health Organization (WHO) or USA CDC criteria, so it would be possible to increase the proportion of indeterminate cases. Second, the specimens analyzed in this study were previously screened as HIV antibodies/antigen reactive by local hospitals or other HIV screening laboratories, thus the detection rate reported here did not reflect the situation of whole population. However, the sample origin had no effect on the proportion of suspected acute/recent HIV-1 infection. Finally, the detection rates of acute and recent HIV infection were roughly estimated based on the WB/RIBA bands, and were not confirmed by other methods, such as BED and NAT. Thus, it is necessary to verify the viral markers of p31, p51, and p66 for recent HIV infection in the future.

In summary, we retrospectively analyzed the WB/RIBA profiles for HIV-1 positive and indeterminate samples during 2012–2018, and found that the suspected recent HIV-1 infection with the absence of p31, p66, and p51 increased in recent years. Comparing the detection between RIBA and WB revealed that RIBA had similar specificity but lower indeterminate results to WB.
